# The construction of CD8+ T cell-associated molecular subtypes in esophageal squamous cell carcinoma reveals tumor heterogeneity, tumor microenvironment, and immunotherapy

**DOI:** 10.3389/fimmu.2026.1775837

**Published:** 2026-03-27

**Authors:** Sinan Cao, Hao Jiang, Yang Li, Ke Yang, Lili Tian, Linwei Li

**Affiliations:** 1 Oncology Department, Henan University People’s Hospital (Henan Provincial People’s Hospital, Zhengzhou University People’s Hospital), Zhengzhou, Henan, China; 2 Oncology Department, The First Hospital of Dalian Medical University, Dalian, Liaoning, China

**Keywords:** CD8+ T cell, esophageal squamous cell carcinoma, heterogeneity, immunotherapy, RhoB, single-cell RNA-seq

## Abstract

**Background:**

Esophageal squamous cell carcinoma (ESCC) is a cancer that is common worldwide. Its morbidity and mortality rates remain high, seriously threatening human health. In the tumor microenvironment (TME), CD8+ T cells undergo a series of dynamic changes that have important implications for tumor progression and the efficacy of immunotherapy. Our research aims to construct novel molecular subtypes through the gene expression profiling of CD8+ T cells during differentiation and to predict the prognostic and therapeutic effects in ESCC patients.

**Methods:**

In the single-cell sequencing (scRNA-seq) analysis, we clustered and visualized cell subsets using the Seurat package, removed batch effects between samples using the Harmony package, and performed pseudo-time analysis of CD8+ T cells using the Monocle2 package. In the bulk-RNA analysis, non-negative matrix factorization (NMF) was applied to construct molecular subtypes, and the unique expression of molecular subtypes in prognostic assessment, biological processes, genomic variation, and immune microenvironment composition was explored. The drug sensitivity of different subtypes was further studied. The causal relationship between gene expression during CD8+ T cell differentiation and ESCC occurrence was inferred using Mendelian Randomization (MR) analysis. In addition, the biological functions of key genes were further explored by *in vitro* cellular experiments.

**Results:**

Based on the genes during CD8+ T cell differentiation, we identified three molecular subtypes with different clinical outcomes. To ensure the robustness and reproducibility of the molecular subtypes, we used the GSE53625 cohort for validation. Among them, the C1 subtype showed higher genomic variation, and its patients had significantly worse prognoses than the C2 and C3 subtypes. It is worth noting that the C3 subtype exhibits an “immune-heat” phenotype, which is accompanied by a large number of immune cell infiltrates, up-regulated immunological checkpoint molecules, and greater sensitivity to immune therapy. In addition, we have screened a series of potential therapeutic drugs, which provides strong support for future clinical translational research. Finally, the results of Mendelian randomization analysis indicated that the RHOB gene could increase the risk of ESCC development. In addition, *in vitro* cellular assays such as CCK-8, scratch assay and Transwell verified that RHOB could promote the proliferation, migration and invasion of ESCC cells *in vitro*.

**Conclusion:**

This study constructed molecular subtypes related to CD8+ T cells and analyzed in depth the biological characteristics, genomic variation, and role of these three molecular subtypes in the tumor microenvironment and immunotherapy. These molecular subtypes allow us to more accurately identify patient groups and thus provide more effective treatment strategies for ESCC patients. In addition, this study identified RHOB as a novel biomarker for esophageal squamous cell carcinoma and verified that RHOB was associated with proliferation, migration and invasion of ESCC by *in vitro* cellular assays.

## 1 Introduction

According to the latest GLOBOCAN 2022 data, esophageal cancer remains a major global health burden, ranking 11th in incidence and 7th in mortality among all cancers. ESCC continues to be the predominant histological subtype, accounting for approximately 85-90% of cases globally (1). The first treatment choice for early ESCC is surgery, but for advanced or metastatic patients, treatment options are limited. Chemotherapy regimens with platinum in combination with paclitaxel/fluorouracil have long been the main first-line treatment option, but their efficacy is limited, and the median overall survival usually does not exceed 12 months (2). Recently, immunotherapeutic strategies have provided a novel direction for the treatment of ESCC, especially in neoadjuvant and first-line treatment in combination with chemotherapy, which has shown significant efficacy (2). However, accurately identifying the patient subgroups suitable for immune checkpoint inhibitors (ICIs) therapy remains a challenge (3), which requires a deep understanding of the heterogeneity of the tumor genome, which is critical for stratifying patients and personalizing treatment.

We can uncover the intricate genetic architecture of malignancies and gain a profound understanding of the expressed gene diversity of tumor tissue thanks to scRNA-seq technologies (4). Compared with traditional sequencing methods, this technology can provide a detailed gene expression map of individual cells, thereby enhancing our understanding of tumor biology (5, 6). TME, a complex ecosystem made up of tumor cells, stromal cells, immune cells, and their secretions, is essential to the growth and spread of tumors (7). The interactions and dynamics of immune cells have a crucial influence on shaping the characteristics of the microenvironment, influencing the tumor immunological response, and determining the clinical outcome of patients (8). T cells are crucial players in the adaptive immune response and are essential for maintaining immune homeostasis, immune surveillance, and tumor control. In tumor immunotherapy, T cells serve as the primary effectors for immune checkpoint blockade therapies, and their activation levels and functions are directly related to treatment efficacy and patient prognosis (9, 10). In untreated ESCC patients, dysfunctional and exhausted T cells exacerbate the immunosuppressive microenvironment in advanced patients (11, 12). T cells can be categorized into CD4+ and CD8+ T cell subsets. Both kinds of cells are essential to the immune response against tumors. However, CD8+ T cells, serving as the pivotal effector lymphocytes in the immune response, are indispensable for the success of anti-neoplastic immunotherapeutic strategies (13). Despite progress in immune-related research, a comprehensive classification based on the dynamic differentiation of CD8+ T cells in ESCC is still lacking. Our central hypothesis is that the differentiation states of CD8+ T cells define distinct molecular subtypes with unique prognostic and therapeutic landscapes. This study aims to answer: How do CD8+ T cells evolve within the ESCC microenvironment? Can differentiation-related genes identify robust ESCC subtypes? and Do these subtypes differ in their response to immunotherapy and chemotherapy?

To address these questions, this study utilized scRNA-seq data to profile immune cells within the neoplastic ecosystem of ESCC patients and to analyze the biological processes of these cells in depth. Through the application of developmental trajectory mapping, we uncovered the evolutionary route of CD8+ T cells toward a functionally exhausted phenotype. Furthermore, we constructed three molecular subtypes based on genes involved in CD8+ T cell differentiation from Bulk-RNA data and validated these subtypes using external datasets to ensure their robustness and reproducibility. We observed significant differences in the three molecular subcategories with respect to prognostic assessment, involved biological processes, genomic variation, and composition of the immunological milieu, and investigated their reactivity to immunotherapeutic interventions and drug sensitivity. In addition, key genes were identified using Mendelian randomization analysis, and the biological functions of the key genes were further explored by *in vitro* cellular experiments.

## 2 Materials and methods

### 2.1 Data acquisition and processing

In this research, we first extracted the scRNA-seq data set GSE188900 from the Gene Expression Omnibus (GEO) database, which covers 7 tumor samples and 1 normal sample. Next, we obtained the transcriptome data, single nucleotide variation (SNV) data, and clinical information of ESCC from the Cancer Genome Atlas (TCGA) database. From the TCGA-ESCA dataset, we selected 95 histologically confirmed ESCC tumor samples and 11 matched normal samples (excluding 78 esophageal adenocarcinoma [EAC] samples based on clinical annotations).In addition, to validate the results, we downloaded the GSE53625 (n = 358) and GSE104958 (n = 46) datasets from the GEO database as independent validation cohorts. To ensure compatibility between the TCGA and GEO data, we log2-transformed the gene expression data for subsequent analysis (**Supplementary Table 1**).

### 2.2 ScRNA-seq data analysis

For scRNA-seq data analysis of ESCC, we processed and integrated data using the Seurat package (14). During the data preprocessing stage, cells with gene counts below 500 and mitochondrial gene expression ratios higher than 15% were eliminated. In addition, genes expressed in at least three cell groups were the only ones we kept, and ultimately we obtained 19,427 single cells. To eliminate batch effects between samples, we performed batch correction using the Harmony algorithm (15). The RunTSNE function was used to display the visualization results of cell clustering. First, we identified the marker genes using the FindAllMarkers function, and then each cell subset was annotated using the singleR package (16). Finally, pseudo-time analysis was conducted using the Monocle2 package to reveal the dynamic changes in cell states and differentiation trajectories (17).

### 2.3 Identification of the ESCC subtype

Based on the pseudotime trajectory of CD8+ T cells, we further identified genes that were significantly differentially expressed along the differentiation trajectory. A total of 51 genes associated with CD8+ T cell developmental dynamics were extracted from the GSE188900 scRNA-seq dataset (**Supplementary Table 2**), which were subsequently used for molecular subtype identification by NMF clustering in bulk RNA-seq data. Specifically, we first set the ranking range to 2 to 9, the number of iterations to 100, and the “lee” algorithm to perform NMF. To determine the optimal number of clusters, we comprehensively considered multiple evaluation indicators such as the cophenetic correlation coefficient, dispersion index, and silhouette coefficient (18). We then used the functions in the survival package to perform a Kaplan-Meier survival analysis and a Cox regression analysis.

### 2.4 Enrichment analysis

First, to find the genes that were differentially expressed for each subtype, we conducted a differential analysis using the limma package. We then ranked these genes in descending order based on their logFC (19). Next, in order to gain a deeper understanding of the biological processes and pathways involved in these differentially expressed genes, we used the clusterProfiler package to perform systematic gene set enrichment analysis (GSEA) of gene sets from the GO database and KEGG database (20).

### 2.5 Mutation analysis

After obtaining somatic variant data in the Mutation Annotation Format (MAF) from the TCGA database, we used the Maftools package to analyze these somatic mutation data. First, the read.maf function was used to read the data, and then the oncoplot function was used to generate a heatmap of mutated genes, showing the pattern of gene mutations in different samples (21). In addition, we performed a copy number variation (CNV) analysis using the cBioPortal database. This step allowed us to visually observe the copy number changes of genes in the sample. The top ten genes with the most homozygous deletion and amplification were of special interest to us since they are probably crucial for the emergence and progression of malignancies (22).

### 2.6 Analysis of the tumor immune microenvironment and immunotherapy

To explore the immune infiltration among different subtypes, we obtained an immune gene set containing 28 immune cell subtypes based on previous research (23) (**Supplementary Table 3**). Then, we used the GSVA package’s single-sample gene set enrichment analysis (ssGSEA) method to measure the relative infiltration levels of these 28 immune cell subtypes in tumor tissues (24). In addition, we systematically evaluated the expression levels of 27 immune checkpoint molecules and 9 human leukocyte antigen molecules, which are essential for immunological responses (25, 26) (**Supplementary Table 4**). To estimate the response of various subtypes to immunotherapy, we used one of the following three techniques. First, we calculated the T cell inflammatory signature (TIS) score, which depends on 18 genes that are closely related to inflammation and obtained through the ssGSEA algorithm. Studies have shown that a higher TIS score often predicts a better response to immunotherapy in patients (27). Second, we examined how various subtypes could react to anti-PD-1 and anti-CTLA-4 therapies using the unsupervised submap approach (28). Finally, we cite the TIDE score, which can visually reflect the degree of tumor immune dysfunction and rejection. Notably, tumors with high TIDE scores often predict that immune checkpoint inhibitor therapy may not be effective (29).

### 2.7 Drug sensitivity analysis and CMap analysis

We utilized the OncoPredict package to compare drug responsiveness in different subgroups and screen for potential targeted therapeutic drugs and chemotherapeutic drugs (30). The genetic profiles of 198 anti-cancer drugs and their genetic profiles were obtained from the Genomics of Drug Sensitivity in Cancer (GDSC) database (31). The Connectivity Map (CMap) discovers connections between diseases, genes, and treatments through cellular responses to perturbations (32). First, we identified differentially expressed genes (150 downregulated genes and 150 upregulated genes) in the three subtypes using the limma package and uploaded them to the Cmap database for analysis using the L1000 platform.

### 2.8 Mendelian randomization analysis

The eQTL data for CD8+ T cell-related genes were obtained from the eQTLGen consortium database (33), which includes 31,684 individuals. Specifically, we used the cis-eQTL summary statistics associated with CD8+ T cells to ensure the relevance of our genetic instruments. To minimize potential bias from population stratification, both the exposure (eQTL) and outcome (ESCC GWAS) datasets were restricted to populations of European ancestry. The outcome data from IEU OpenGWAS (ID: ebi-a-GCST90018841) predominantly consists of individuals from the UK Biobank (34). We used the “TwoSampleMR” package for MR analysis to infer causal relationships between exposure and disease outcomes (35). The causal relationship between CD8+ T cell-related genes and esophageal cancer was assessed using Inverse-Variance Weighting and Wald ratio methods.

### 2.9 Cell culture and siRNA transfection

ESCC cell lines KYSE150 and TE-1 were from the Chinese Academy of Sciences cell bank. Cells were cultured in RPMI-1640 medium with 10% FBS and 1% penicillin/streptomycin at 37 °C in a 5% CO_2_ incubator. All cells passed mycoplasma testing and STR authentication. RHOB -targeting siRNA and negative control were synthesized by Shanghai HANHUI Biological Technology Co., Ltd., and transfected using Lipofectamine™ RNAiMAX reagent as instructed. The siRNA sequences are shown in **Supplementary Table 5**.

### 2.10 Quantitative real-time PCR

qRT-PCR was used to detect knockdown efficiency in tumor cells. Total RNA was extracted from ESCC cells using TRIzol (Ambion), RNA concentration was measured by a micro - spectrophotometer, and samples were stored at - 80 °C. RNA was reverse - transcribed to cDNA using PrimeScript™ RT Kit (with gDNA Eraser), and qRT-PCR amplification was performed using TB Green Premix Ex Taq™ II (Takara). GAPDH was used as the reference gene. Primer sequences are shown in **Supplementary Table 5**.

### 2.11 CCK-8 assay

The CCK-8 assay detects cell proliferation. Seed 1×10_4_ cells per well in a 96 - well plate. At time points (0h, 24h, 48h, 72h), replace the medium with 100 μL CCK-8 solution (9:1 medium:CCK-8), incubate for 2 hours away from light. Measure absorbance at 450nm using a microplate reader. Plot a proliferation curve by comparing OD values over time.

### 2.12 Transwell assay

For the cell migration assay, digest and centrifuge cells, then resuspend them in serum - free medium at 2.5×10^5^/mL. Add 200 μL to the upper chamber and 700 μL of complete medium with 10% serum to the lower chamber. Incubate for 24 hours. Remove the upper medium, wash with PBS, fix with 4% paraformaldehyde for 30 minutes, stain with 0.5% crystal violet for 8 minutes, and analyze cell numbers using a microscope and ImageJ software. For the invasion assay, pre - treat with Matrigel, adjust cell concentration to 5×10^5^/mL, and follow the same steps as the migration assay.

### 2.13 Wound healing assay

When cells are in the logarithmic growth phase, trypsinize and resuspend them. Seed 2×10^6^ cells per well in a 6 - well plate with high - glucose DMEM. Incubate until confluence reaches 90% - 100%. Create a scratch with a 200 μL pipette tip, rinse with PBS, and add 1 mL of medium with 2% serum. Photograph the same field at 0h and 72h under a microscope to assess cell migration.

### 2.14 Statistical analysis

Differences between two groups were compared using a t-test, while those among three groups were compared using the Kruskal-Wallis test. Survival differences among subtypes were analyzed by Kaplan-Meier survival analysis, with the log-rank test used for inter-group comparisons. All analyses were performed using R (version 4.3.1) and GraphPad Prism (version 8.0.2). A two - sided P - value of less than 0.05 was considered statistically significant. (*P < 0.05; **P < 0.01; ***P < 0.001; ****P < 0.0001).

## 3 Results

### 3.1 Single-cell atlas of immune cells

**Figure 1:**
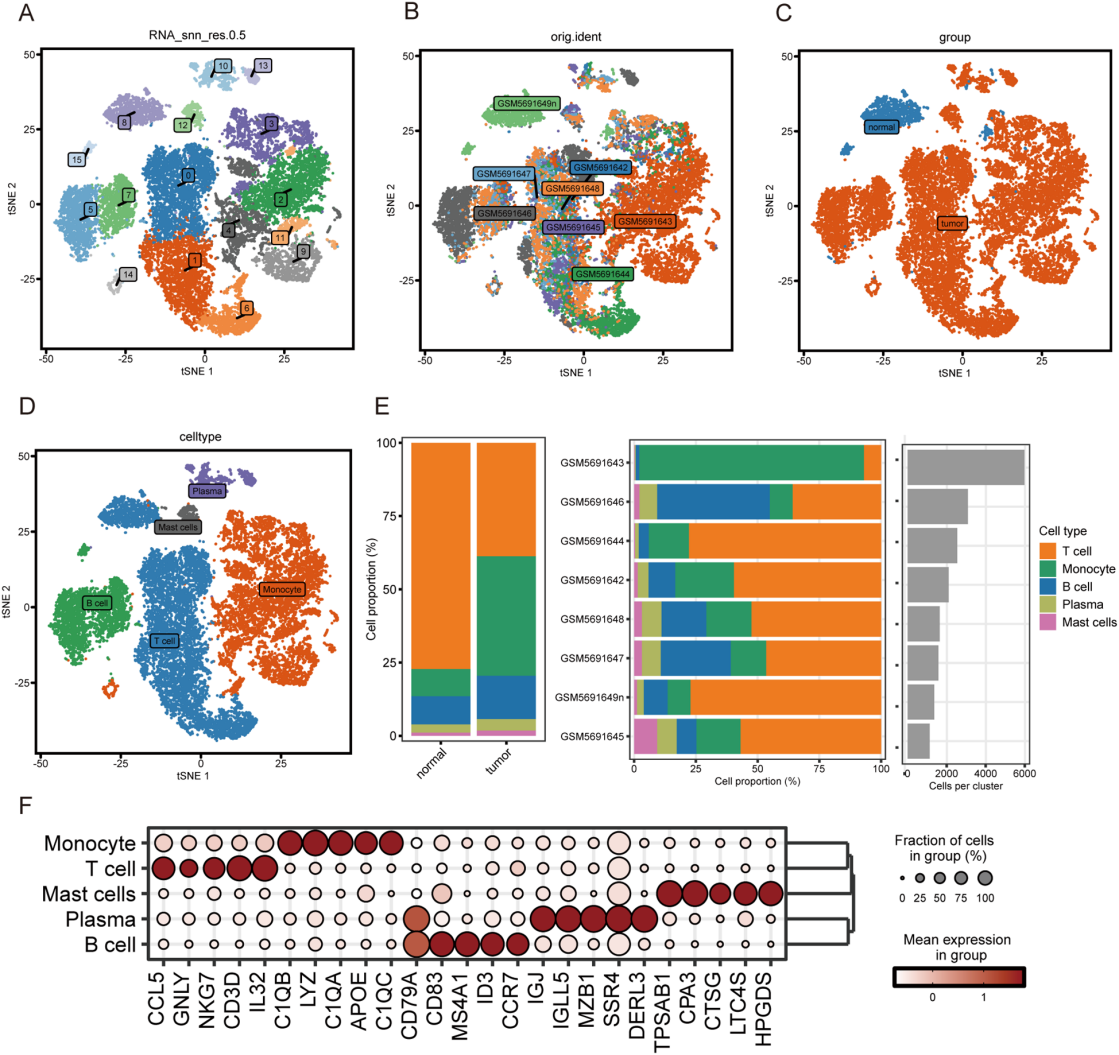
Immune cell profiles from tumor tissue and normal tissue. **(A)** 16 cell clusters of t-SNE. **(B)** t-SNE for 7 samples. **(C)** t-SNE of normal and tumor samples. **(D)** t-SNE of five immune cells. **(E)** Proportions of five immune cell types in tumor versus normal tissues (left) and across individual samples (middle). The rightmost bar plot shows the total absolute number of cells for each identified immune cell type across the entire dataset. **(F)** Bubble diagram showing the top five marker genes for each of the five immune cell types.

First, we eliminated low-quality cells from the GSE188900 single-cell dataset by performing strict quality control and ultimately included 19,427 high-quality immune cells for subsequent analysis. These immune cell populations were identified and visualized by t-SNE clustering using the Seurat package for dimensionality reduction and unsupervised clustering. 19,427 immune cells from esophageal squamous cell carcinoma tissue and normal tissue were grouped into 16 subgroups (**Figure 1A**). **Figure 1B** shows the dimensionality reduction clustering map of single cells from different samples. **Figure 1C** displays the clustering map of single cells from tumor and normal tissues with reduced dimensionality. According to the SingleR package and specific cell biomarkers, these immune cells can be divided into five immune cell subsets, including B cells (CD19, MS4A1), plasma cells (MZB1, IGJ), monocytes (CD68, CD14), mast cells (TPSAB1, KIT), and T cells (CD3D, CD3E) (**Figure 1D**). The distribution of these signature genes in immune cell subsets is shown in the figure (**Supplementary Figures 1A–E**). The percentage of five immune cells in tumor and normal tissue, as well as the variations in these five immune cell proportions between samples, are graphically shown in **Figure 1E**. Among the five immune cell subsets, T cells account for the largest proportion. B cells and monocytes follow. In contrast, the proportion of mast cells is relatively small. **Figure 1F** shows the top 5 genes expressed by the five immune cell types. We made the decision to learn more about the biological roles of T cells, as they are essential for identifying and destroying tumor cells in addition to being involved in adaptive immune responses.

### 3.2 Pseudotime analysis of CD8+ T cells

**Figure 2:**
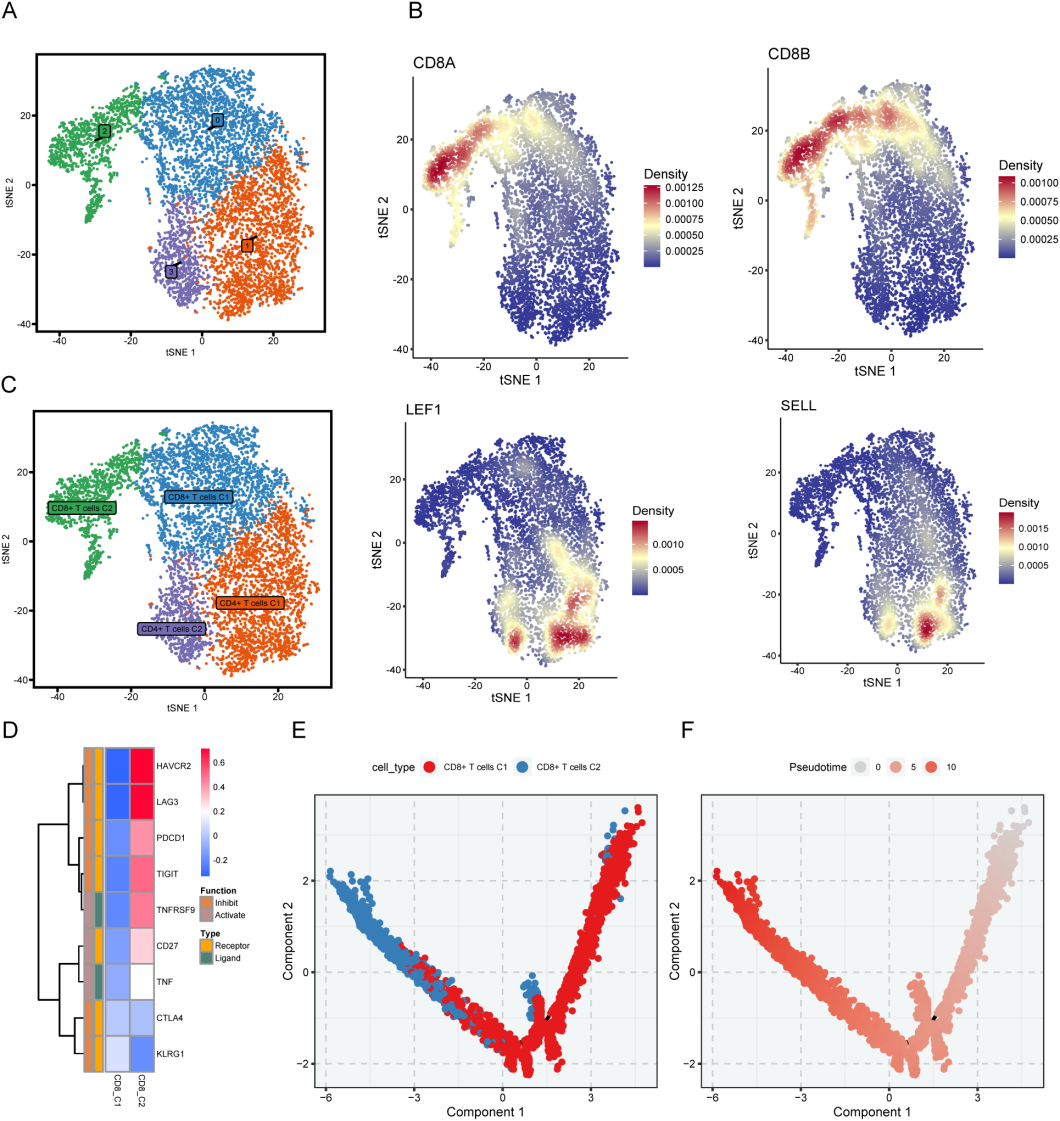
Mapping of T cells and proposed time-series analysis of CD8+ T cells. **(A)** t-SNE plots of the four T cell subtypes. **(B)** Characterization plot of CD8A, CD8B, LEF1 and SELL gene expression. **(C)** t-SNE plots of CD4+ T cells and CD8+ T cells. **(D)** Heat map of immune checkpoint expression in CD8+ T cells. Pseudotemporal analysis of CD8+ T cells:**(E)** Colored according to cell type, **(F)** Colored according to developmental time.

To thoroughly characterize the CD8+ T cell development trajectory in tumor tissue, we used the Monocle2 tool for cell trajectory analysis. First, we performed further dimensionality reduction and clustering on the T cells in ESCC tissue, successfully identifying four different T cell subtypes (**Figure 2A**). According to previous studies, we discovered that the LEF1 and SELL genes were highly expressed in CD4+ T cells, while CD8+ T cells were significantly highly expressed the CD8A and CD8B genes (36). The gene expression patterns on the t-SNE plot clearly show that the characteristic genes of CD4+ T cells are highly expressed in clusters 1 and clusters 3, while the characteristic genes of CD8+ T cells dominate in clusters 0 and clusters 2 (**Figure 2B**). Based on these findings, we designated CD4+ T cells in clusters 1 and 3 as C1 and C2, respectively. Additionally, CD8+ T cells C1 and C2 are seen in clusters 0 and 2, respectively (**Figure 2C**). It is worth noting that in CD8+ T cells C2, we found that multiple immune checkpoint molecules were highly expressed, including HAVCR2, LAG3, TIGIT, PDCD1, and TNFRSF9 (**Figure 2D**). According to previous research reports, high expression of these immune checkpoint molecules is a typical feature of exhausted CD8+ T cells (37). Therefore, our hypothesis is that CD8+ T cells C2 can be a collection of worn-out CD8+ T cells. The results of the proposed time-series analysis further revealed the dynamic process of CD8+ T cell differentiation to an “exhausted” state in the TME (**Figures 2E, F**). Earlier studies have verified that the functional loss of exhausted CD8+ T cells is one of the important mechanisms by which tumor achieve immune escape (38). Therefore, we think that the genes governing CD8+ T cell development could develop into possible biomarkers for estimating the prognosis of individuals with ESCC.

### 3.3 NMF identifies molecular subtypes associated with CD8+ T cells

**Figure 3:**
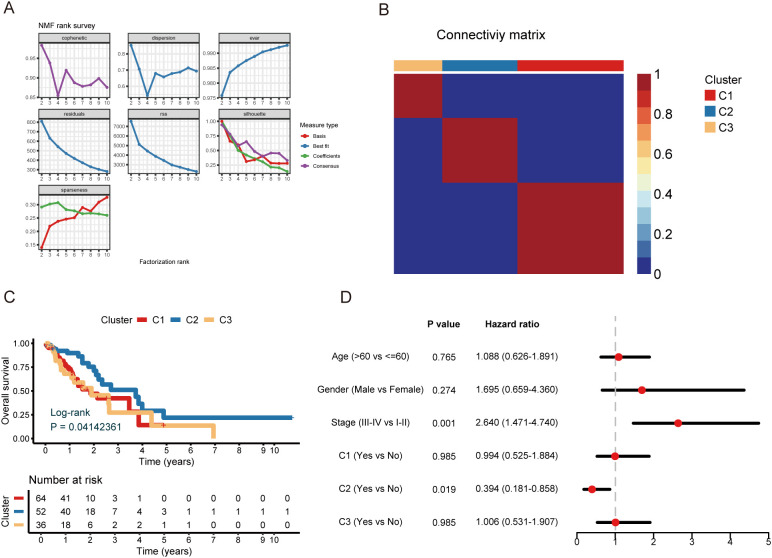
Three molecular isoforms were constructed by NMF.**(A)** NMF rank and phenotypic correlation coefficient with an optimal rank of 3. **(B)** Heat map of the NMF consensus matrix. **(C)** Kaplan-Meier survival curves for ESCC patients in the TCGA dataset. **(D)** Multivariate Cox regression for ESCC patients in the TCGA dataset.

To further translate the dynamic transcriptional changes of CD8+ T cells into clinically relevant stratification, we extracted 51 genes that were significantly associated with the CD8+ T cell differentiation trajectory from the GSE188900 scRNA-seq dataset (**Supplementary Table 2**). These genes were subsequently used for NMF clustering analysis in the TCGA bulk RNA-seq cohort to construct CD8+ T cell-associated molecular subtypes. We performed a comprehensive cluster analysis of the TCGA cohort using the NMF algorithm and genes associated with CD8+ T cells. Based on a comprehensive consideration of the phenotype factor and contour score, we determined that the optimal number of clusters was 3 (**Figure 3A**). Accordingly, three subgroups of the TCGA cohort were precisely identified: 64 samples were found in Cluster 1, 52 samples were found in Cluster 2, and 36 samples were found in Cluster 3. The consensus matrix heatmap shows clear boundaries between the three clusters, which is strong evidence for the accuracy and robustness of the clustering results (**Figure 3B**). We conducted a survival study to look more closely at these subgroups’ prognostic significance. The findings demonstrated that the overall survival (OS) of individuals with C1 and C3 subtypes was comparatively low, while patients with C2 subtype showed a good prognosis (*P* < 0.05) (**Figure 3C**). Subsequently, we also used multivariate Cox regression analysis, and the findings make it abundantly evident that the C2 subtype is a predictor of outcome (**Figure 3D**). To confirm the reliability and robustness of the clustering results, we validated them in the GSE53625 cohort. Notably, in the GSE53625 cohort, we also came to the conclusion that three clusters was the ideal quantity. In addition, the results of a survival analysis of this cohort were consistent with previous studies, and multivariate Cox regression analysis once again confirmed the status of subtype C2 as an independent prognostic factor (**Supplementary Figures 2A–D**).

### 3.4 Enrichment analysis

**Figure 4:**
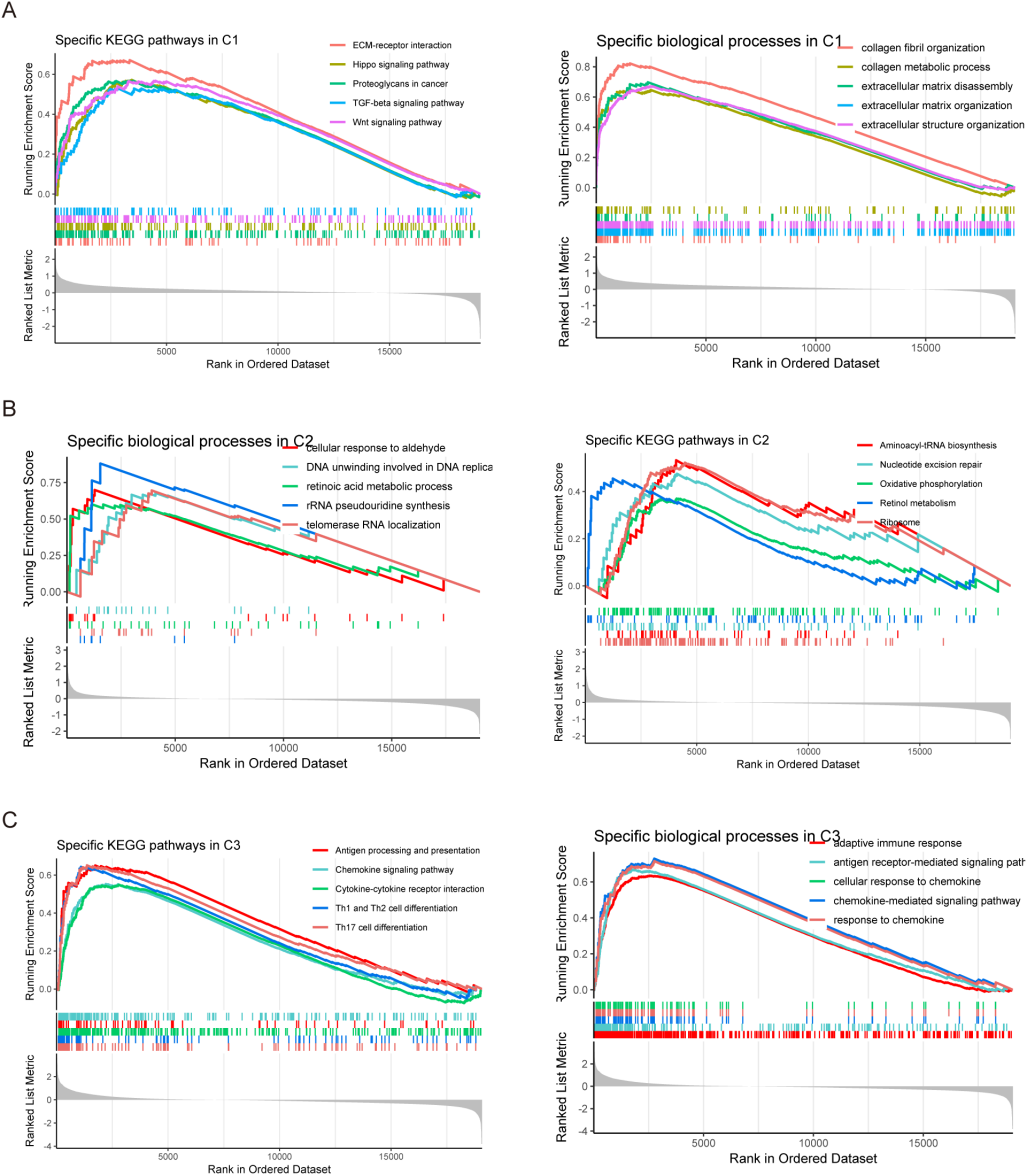
Enrichment analysis of three molecular isoforms. **(A)** Enrichment analysis of GO and KEGG gene sets of C1 isoforms. **(B)** Enrichment analysis of the GO and KEGG gene sets of the C2 isoform. **(C)** Enrichment analysis of GO and KEGG gene sets of C3 isoforms.

To further comprehend how the three subtypes’ biological functions differ from one another, we adopted a series of systematic bioinformatics analysis methods. First, using the limma package for differential analysis, we accurately identified the differentially expressed genes for each subtype. We then performed GSEA analysis on these differentially expressed genes using gene sets from the GO database and KEGG database. The results of the study showed that the C1 subtype was significantly enriched in key areas of biological processes such as collagen fibrous tissue, extracellular matrix tissue, extracellular structural tissue, extracellular matrix disassembly, and collagen metabolic processes. In the KEGG pathway analysis, the C1 subtype was significantly enriched in extracellular matrix receptor interactions, proteoglycans in cancer, TGF-beta, Wnt, and Hippo signaling pathways, respectively, which are crucial to the growth and genesis of tumors (**Figure 4A**). In contrast, the C2 subtype is significantly enriched in biological processes such as pseudouridine synthesis of rRNA, RNA localization of telomerase, retinol metabolic processes, cellular response to aldehydes, and DNA unwinding involved in DNA replication. In the KEGG pathway analysis, the C2 subtype was significantly enriched in pathways such as ribosomes, aminoyl-tRNA biosynthesis, nucleotide excision repair, retinol metabolism, and oxidative phosphorylation. These pathways are closely related to the cell’s metabolic, proliferative, and repair functions (**Figure 4B**). The C3 subtype is significantly enriched in key biological processes such as adaptive immune responses, antigen receptor-mediated signaling, chemokine-mediated signaling, chemokine responses, and cell responses to chemokines. In KEGG pathway analysis, Th17 cell differentiation, Th1 and Th2 cell differentiation, cytokine-cytokine receptor interactions, antigen processing and presentation, and chemokine signaling pathways are all markedly enhanced in the C3 subtype (**Figure 4C**). These pathways are essential for both the inflammatory and immunological responses. As mentioned above, through GSEA analysis, we revealed differences in the molecular mechanisms and pathways of the three subtypes in terms of biological function. This analysis provides an important theoretical basis for helping us understand the functional heterogeneity between subtypes.

### 3.5 Gene mutation analysis

**Figure 5:**
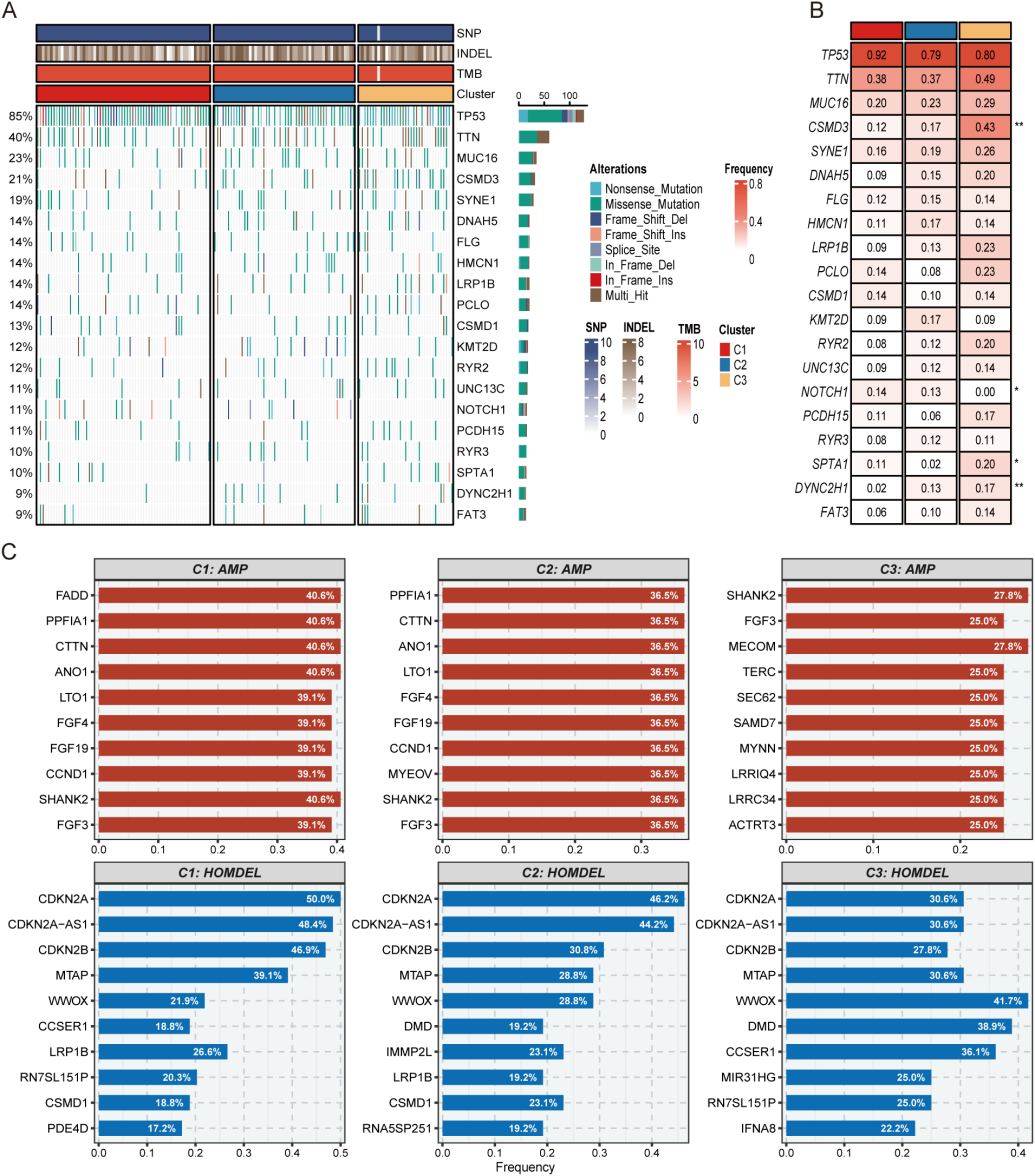
Mutation analysis of the three molecular isoforms. **(A)** Waterfall plot of the top 20 frequently mutated genes in the TCGA dataset. **(B)** The top 20 frequently mutated genes of the three molecular subtypes. **(C)** The first 10 genes amplified and genes missing in the purities of the three molecular isoforms. *p < 0.05, **p < 0.01.

We examined the TCGA cohort’s somatic mutation data using the Maftools package. First, we displayed the twenty of the most commonly mutated genes (**Figure 5A**), among which TP53 and MUC16 are widely confirmed to be closely related to tumorigenesis (39, 40). Next, we compared the differences in frequently mutated genes among the three subtypes (**Figure 5B**). It was found that mutations in TTN and MUC16 were more prominent in C3, while TP53 mutations dominated in C1. According to earlier research, cancer can result from the slow accumulation of genetic mutations (41). Compared with other subgroups, patients in the C2 subgroup showed a lower frequency of gene mutations, which further implies that patients in the C2 subgroup would have a better prognosis and is in line with the findings of our earlier prognostic study. To investigate the features of chromosomal variation across several subtypes in greater detail, we used the cBioPortal database to conduct an in-depth analysis of copy number variation in the three subtypes and summarized the top 10 gene amplifications and homozygous deletions (**Figure 5C**). It’s important to note that patients in the C1 subgroup had more copy number variation, and among all subtypes, the CDKN2A gene exhibited the most significant homozygous deletion (**Figure 5C**). According to earlier studies, ESCC formation and immune escape are induced when the tumor suppressor gene CDKN2A is lost (42). Taken together, patients in C1 exhibited significant genomic variation, indicating a high degree of genomic instability.

### 3.6 Immune infiltration analysis and immunotherapy evaluation

**Figure 6:**
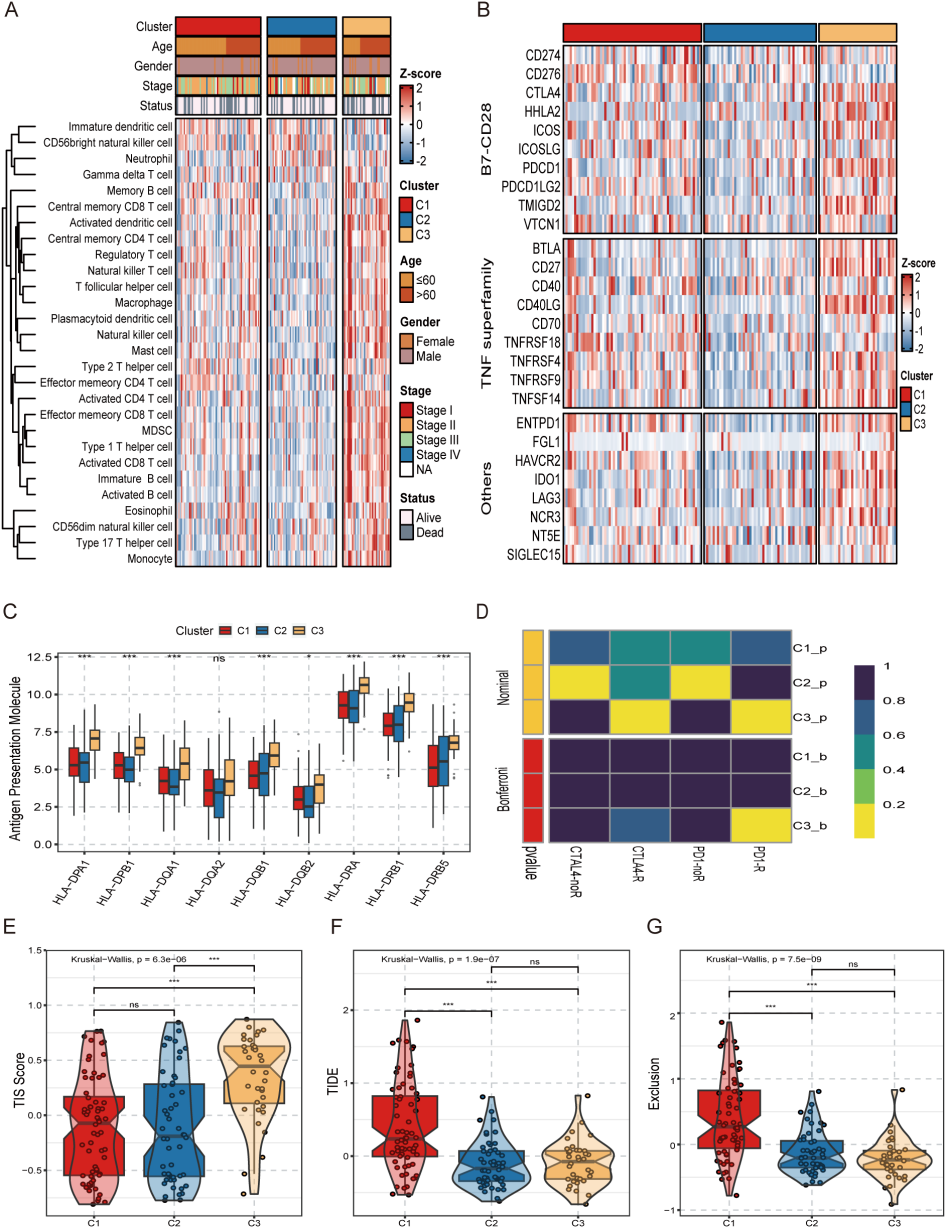
Analysis of differences in tumor infiltrating immune cells and immunotherapy between the three molecular subtypes. **(A)** Heatmap of the infiltration abundance of 28 immune cells of three molecular subtypes. **(B)** Heatmap of molecular expression of immune checkpoints for three molecular subtypes. **(C)** Comparative graph of the grouped expression of human leukocyte antigen molecules of the three molecular subtypes. **(D)** SubMap analysis of three molecular subtypes. **(E)** T-cell inflammatory signature scores for three molecular subtypes. **(F)** TIDE scores for the three molecular subtypes. **(G)** Exclusion scores for the three molecular subtypes.^ns^*P* > 0.05, ***P < 0.001.

We selected a gene set of 28 immune cells that had been identified in earlier research to better examine the variations in immune infiltration among the three subtypes. We then employed ssGSEA analysis to precisely determine the relative abundance of different immune cell types within tumor tissue. The study revealed a striking phenomenon: compared with other subgroups, the C3 subtype showed a richer immune cell infiltration (**Figure 6A**). This finding classifies C3 patients as an “immune hot” subtype, with their tumor microenvironment being densely populated with various immune cells such as memory B cells, activated B cells, activated dendritic cells, CD4+ T cells, and CD8+ T cells (*P* < 0.05) (**Supplementary Figure 3A**). Furthermore, based on the expression of immunological checkpoint molecules, we compared the traits of the three subtypes. The results showed that the C3 subtype expressed higher levels of immune checkpoint molecules, including PDCD1 (PD-1), CTLA-4, and LAG3, compared to the other subtypes, it would suggest that patients with the C3 subtype are more vulnerable to immunotherapy (**Figure 6B**). In addition, the expression of human leukocyte antigen (HLA) molecules was significantly higher in C3 subtype patients, which further suggests that the immune activation state may be more intense in the TME of these patients (**Figure 6C**). We employed the TIS, TIDE, and Submap methodologies to assess the three subtypes’ individual responses to immunotherapy. Patients with the C3 subtype may respond well to anti-PD-1 treatment, according to the Submap analysis’s findings (Bonferroni corrected *P*-value *P* < 0.05) (**Figure 6D**). The TIS analysis also showed that patients with the C3 subtype had higher TIS scores, which indicates that they may derive more clinical benefits from immune checkpoint inhibitor therapy (**Figure 6E**). Meanwhile, to assess the possible effectiveness of immunotherapy in the three subtypes, we employed the TIDE method. The findings indicated that subtype C1 had a considerably higher TIDE score and Exclusion score, indicating that immune evasion may be more prevalent in this subtype (**Figures 6F, G**). We also performed the same immunotherapy evaluation in the GSE53625 dataset and obtained the same results (**Supplementary Figures 3B–D**). In summary, our study clearly shows that patients with the C3 subtype respond more positively to immunotherapy. This finding provides a strong scientific basis for individualized immunotherapy strategies for ESCC patients in clinical practice.

### 3.7 Screening for small molecules and drug sensitivity analysis

**Figure 7:**
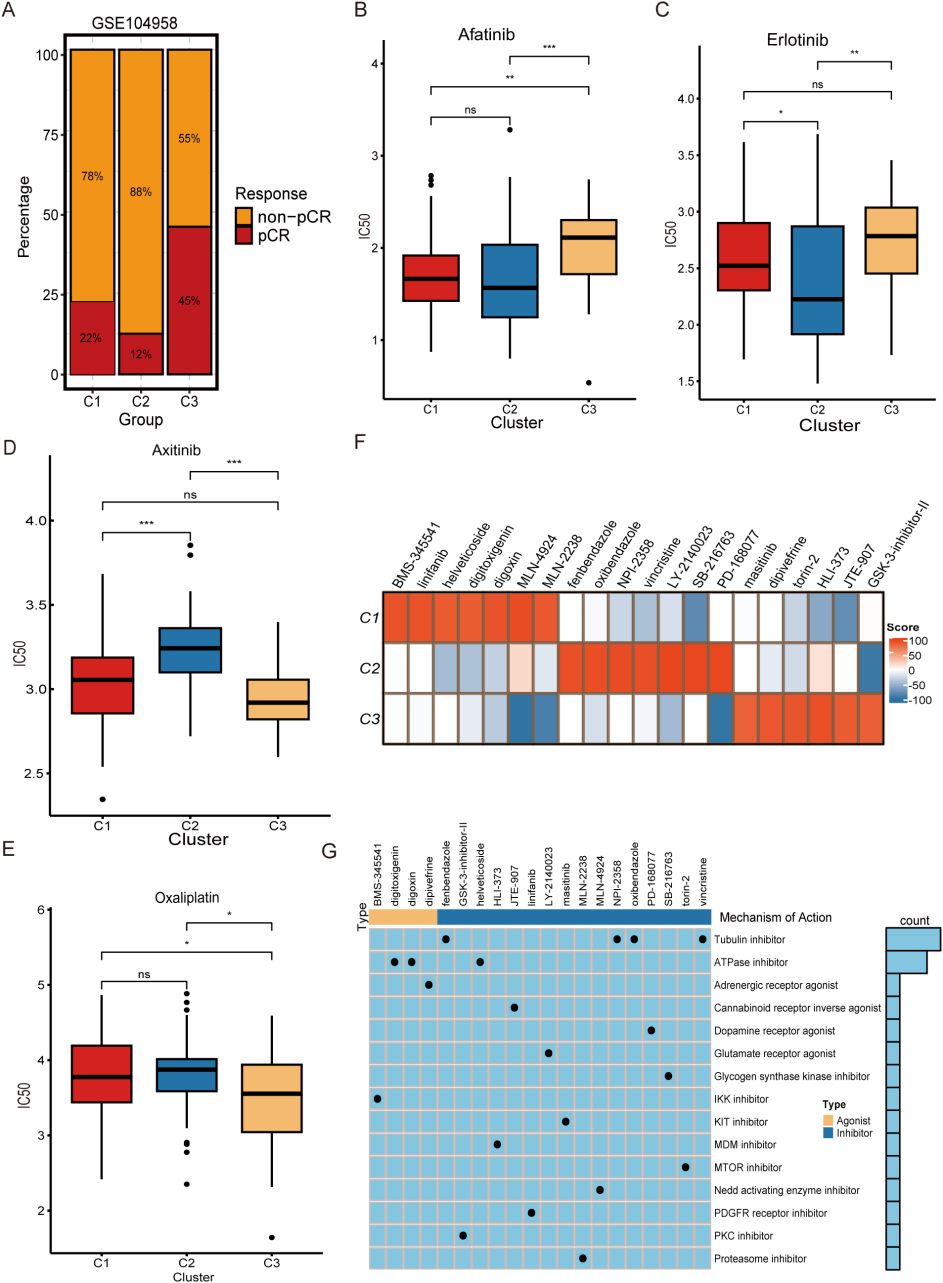
DCF therapy evaluation, drug sensitivity analysis, and small molecule drug screening. **(A)** Treatment response ratios for the three molecular subtypes in the GSE104958 cohort. IC50 box plots for **(B)** Afatinib, **(C)** Erlotinib, **(D)** Axitinib, and **(E)** Oxaliplatin. **(F)** Heat map of three molecular subtypes of candidate small molecule drugs. **(G)** Mechanisms of action of candidate small molecule drugs.^ns^*P* > 0.05, *P < 0.05, **P < 0.01, ***P < 0.001.

We collected data from the clinical treatment cohort GSE104958. DCF therapy (docetaxel/cisplatin/fluorouracil) is widely used in clinical practice, especially in ESCC patients aged 75 and under. Neoadjuvant DCF therapy has been found to significantly improve survival in patients who undergo surgical resection (43). Our results show that patients with the C3 subtype have an ideal response to DCF therapy (**Figure 7A**). We predicted the sensitivity of tumor cells to various medicines using the OncoPredict software; more drug sensitivity is indicated by lower IC50 values. Olaparib, osimertinib, and rapamycin were found to be highly sensitive in C1 subtype patients (**Supplementary Figures 3E–G**). C2 subtype patients appeared to respond more significantly to afatinib and erlotinib (**Figures 7B, C**). In addition, C3 subtype patients showed higher sensitivity to acetylatinib and osimertinib (**Figures 7D, E**). To screen for candidate small-molecule drugs for the three subtypes, we uploaded the differentially expressed genes (150 downregulated genes and 150 upregulated genes) of the three subtypes to the CMap database. As shown in **Figures 7F**, the candidate small molecule drugs for subtype C1 are MLN-4924, linifanib, BMS-345541, digoxin, MLN-2238, helveticoside, and digitoxigenin; the candidate small molecule drugs for subtype C2 are LY-2140023, PD-168077, SB-216763, NPI-2358, oxibendazole, vincristine, and fenbendazole; and the C3 subtype candidate small molecule drugs are HLI-373, torin-2, dipivefrine, JTE-907, GSK-3-inhibitor-II, and masitinib. In addition, we also show the targeted pathways of these candidate small-molecule drugs, which can be used to develop multiple drugs (**Figure 7G**). These results provide potential therapeutic targets for personalized medicine to achieve more precise treatment strategies.

### 3.8 MR analysis

We performed a Mendelian randomization analysis to investigate the causal association between CD8+ T-cell-related gene expression and ESCC risk. Forest plots showed that genetically predicted higher expression levels of RHOB, HLA-DRB1, IL7R, and APOBEC3G were causally associated with an increased risk of ESCC (*P* < 0.05) (**Supplementary Figure 4**). All chosen instrumental variables exhibited F-statistics greater than 10, indicating the absence of weak instrument bias. Overall, these results suggest that CD8+ T cell-related genes may have an important genetic influence in the pathogenesis of esophageal cancer.

### 3.9 RHOB was able to promote the proliferation, migration and invasion of ESCC cells *in vitro*

**Figure 8:**
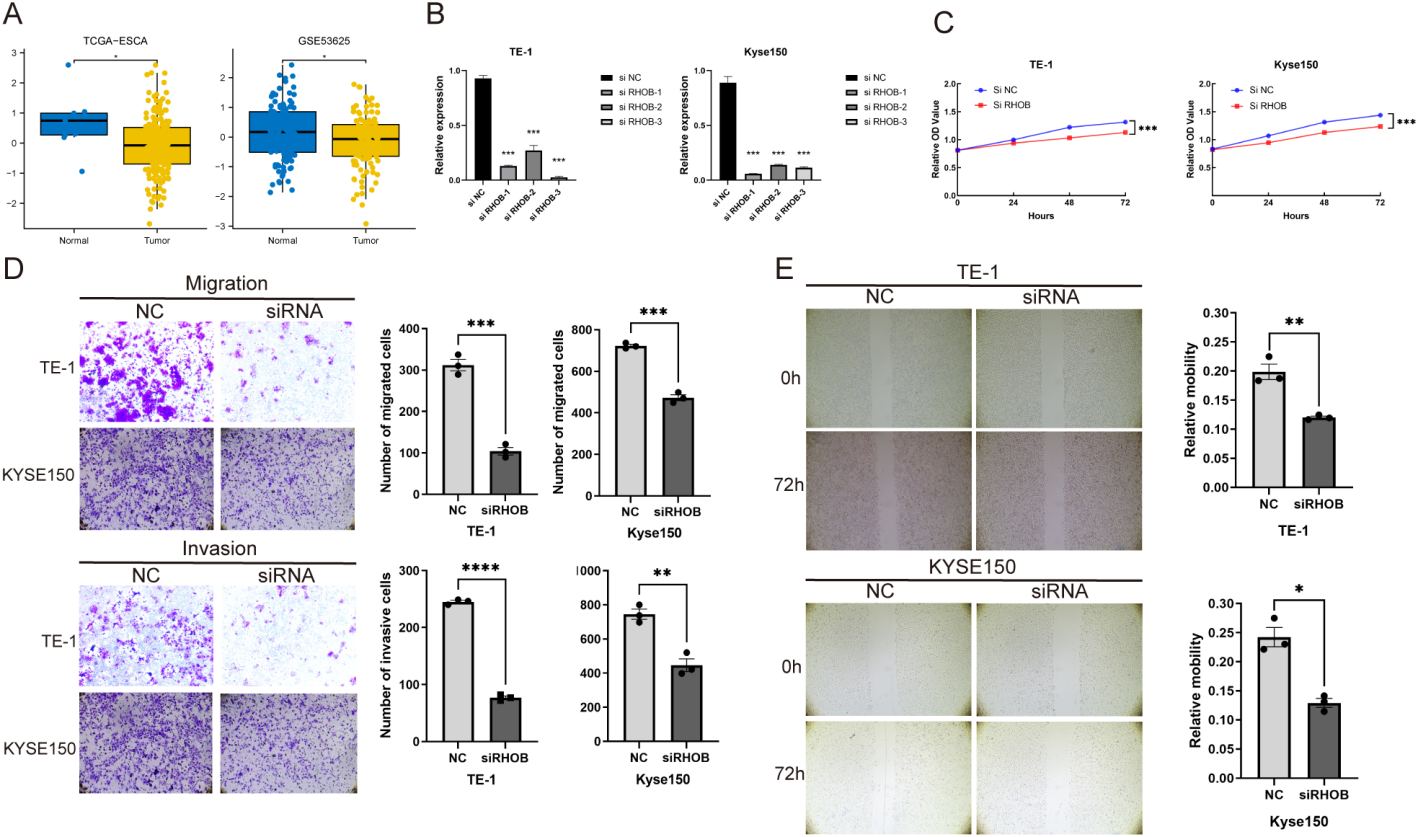
Effect of RHOB on proliferation, invasion and migration of ESCC. **(A)** Expression levels of RHOB in ESCC tissues. **(B)** Validation of RHOB knockdown efficiency. **(C)** CCK-8 assay to detect the proliferation of TE-1 and KYSE150 cells after RHOB knockdown. **(D)** Transwell assay was used to assess the migration and invasion ability of TE-1 and KYSE150 cells after RHOB knockdown. **(E)** Scratch assay was used to detect the effect of RHOB knockdown on the migration ability of ESCC cells. *P < 0.05,**P < 0.01,***P < 0.001,****P < 0.0001.

Based on the MR results indicating that higher RHOB expression is a causal risk factor for ESCC, we further assessed the role of RHOB in ESCC. In the TCGA-ESCA and GSE53625 cohorts, the expression of RHOB was lower in ESCC tissues than in normal tissues (**Figure 8A**). To validate whether reducing RHOB expression could suppress the ESCC oncogenic phenotype as predicted by our MR model, we transfected three si-RHOB into TE-1 and KYSE150 cells to knock down RHOB expression. The validity of RHOB knockdown was verified by qRT-qPCR analysis (**Figure 8B**).First, the cell proliferation ability was assessed by CCK-8 assay. The results showed that the relative cell proliferation capacity of TE-1 and KYSE150 cells was significantly lower than that of the control group after knocking down RHOB (**Figure 8C**). In addition, to verify the effect of RHOB on ESCC cell migration and invasion, we performed Transwell migration and invasion assays, and the migration and invasion abilities of ESCC cells in the NC group were significantly stronger than those of ESCC cells after knockdown (**Figure 8D**). Then, we also performed cell scratch assays and obtained similar results (**Figure 8E**). In summary, we concluded that RHOB could promote the migration, invasion and proliferation of ESCC cells *in vitro*.

## 4 Discussion

Due to its high mortality rate and global prevalence, ESCC is a significant public health concern (44). Although traditional treatments such as surgery, chemotherapy, radiotherapy, and limited targeted therapies have to some extent helped patients live longer, the efficacy of these treatments is still significantly limited due to the complexity and high heterogeneity of ESCC itself (44). The development of immunotherapy in recent years has reenergized the ESCC treatment sector. This innovative therapy has shown extraordinary potential in improving treatment response rates and prolonging patient survival, bringing new hope for ESCC patients (2). However, despite the remarkable progress made in immunotherapy, there are still many pressing challenges to be overcome in order to achieve precision treatment for ESCC. Among these, the difficulty of early diagnosis is particularly prominent. Since ESCC often lacks obvious symptoms in the early stages, many patients are already in the middle and late stages of the disease when diagnosed, thus missing the best opportunity for treatment. In addition, the diversity of postoperative complications is also an important challenge in the treatment of ESCC (45). There are significant differences in the type and severity of postoperative complications between patients, which undoubtedly increases the complexity and uncertainty of treatment. Therefore, in order to meet these challenges, it is particularly important to conduct in-depth molecular typing of ESCC and achieve precision treatment. Molecular typing allows us to better understand the pathophysiology and biology features of ESCC, so as to develop more personalized treatment regimens, with a view to providing patients with more effective treatment options and better survival prognoses.

TME is a complex ecosystem comprising multiple cell types, signaling molecules, and structural components that impair antitumor immune responses by promoting tumor cell growth, activating chronic inflammation, and immunosuppressive mechanisms (46). Immune cells, particularly T cells, are essential for controlling the anti-tumor immune response in TME. Because of their special regulatory role, CD4+ T cells, as helper T cells, are essential to the anti-tumor immune response, which can enhance or inhibit the activity of other immune cells. However, as cytotoxic T lymphocytes, CD8+ T cells play a crucial role in carrying out the anti-tumor immune response by directly destroying tumor cells (47, 48). It is worth noting that CD8+ T cells often undergo a series of dynamic changes during tumor progression, including adverse states such as exhaustion, and these changes may directly affect the exertion of their anti-tumor function. Therefore, an in-depth exploration of the evolutionary trajectory of CD8+ T cells and the molecular mechanisms behind it is crucial for comprehending the immune escape mechanism of ESCC and creating successful treatment plans.

In subsequent analyses, using NMF, three strong molecular subtypes were found. The prognosis for the C2 subtype was better, and it may be used as a stand-alone prognostic predictor, according to additional prognostic analysis. The reliability and stability of the molecular subtypes were then confirmed using the GSE53625 cohort, which produced identical outcomes, verifying the molecular subtypes’ stability and dependability. The possible biological traits of these three subgroups are likewise thoroughly described by our study. The pathways significantly enriched in the C1 subtype are mainly related to the organization, metabolism, and signaling of the extracellular matrix. These pathways are crucial for preserving tissue integrity, controlling cell activity, and contributing to the initiation and spread of cancer. The pathways of the C2 subtype are involved in metabolic reprogramming, maintenance of DNA stability, cell response, and protein synthesis. The C3 subtype’s pathways have a role in immune cell activation, migration, and differentiation. In addition, each of these three groupings has unique genetic traits. The C1 subtype has the most genomic instability, according to somatic mutation and CNV studies. According to earlier research, patients with TP53 mutations are easier to escape from the immune system and have a poorer prognosis (42). Our research also demonstrated that the prognosis is worse for the C1 subtype, which has the largest frequency of TP53 mutations. In addition, deletion of CDKN2A is associated with immune escape in ESCC (42). Accordingly, the higher frequency of CDKN2A mutations in the C1 subtype may indicate that these patients do not have a good response to immuno-therapy. The C3 subtype has stronger immune and inflammatory activity, and these patients may be more inclined to exert antitumor immune activity and benefit from immunotherapy.

Additionally, we examined the infiltration of immune cells in different subtypes and found that memory B cells, CD4+ T cells, CD8+ T cells, activated B cells, and activated dendritic cells were all highly infiltrated in the C3 subtype. Additionally, PDCD1, CTLA-4, and LAG3, three immune checkpoint molecules, were shown to be substantially expressed in the C3 subtype. In addition, the C3 subtype also showed strong antigen presentation capabilities. Immunotherapy efficacy was predicted for different subsets using TIS, TIDE, and Submap methods, and the results showed that the C3 subset may benefit more from immunotherapy. Therefore, it is recommended that patients with the C3 subset should be given priority for immunotherapy. The classification of ESCC into three molecular clusters based on CD8+ T cell differentiation trajectories resonates with the well-established “immune landscape” across various solid tumors. Our C3 subtype mirrors the “Immune-Inflamed” or “Hot” tumor phenotype (49, 50) observed in melanoma and lung adenocarcinoma, where dense T-cell infiltration and high antigen presentation correlate with superior responses to immune checkpoint inhibitors. Conversely, the C1 subtype exhibits characteristics of “Immune-Excluded” tumors, a pattern frequently documented in gastric cancer (51). In such cases, despite the presence of oncogenic signaling, the dense extracellular matrix and activated TGF-beta pathways—which were significantly enriched in our C1 cohort—act as physical and biochemical barriers that sequester T cells in the stroma, preventing them from attacking tumor cells (47). This comparative analysis underscores that while ESCC has unique drivers like TP53 and CDKN2A mutations, its immune evasion mechanisms share fundamental commonalities with other gastrointestinal malignancies, supporting the potential for cross-cancer therapeutic strategies. We identified potential therapeutic drugs for the C1 subtype, such as olaparib and rapamycin. In ESCC, the combination of rapamycin and PLK1 inhibitors in particular showed a stronger anti-tumor effect (44). Using the Camp database, we screened for candidate small-molecule drugs against different subtypes and detailed their targeted pathways, which can be used to develop multiple drugs.

We identified a causal link between CD8+ T cell trajectory genes and ESCC using Mendelian randomization, finding RHOB to be an ESCC risk factor (52). RHOB, a key member of the Ras superfamily, has its activity tightly regulated by guanine nucleotide exchange factors and GTPase - activating proteins. The Rho family of GTPases has 20 members divided into 8 subgroups (53), with RHOA, RHOB, and RHOC being core but functionally distinct members. RhoA and RhoC often act as oncogenes by promoting tumor progression in various cancers. In contrast, RHOB has a dual nature, acting as both an oncogene and a tumor suppressor in different cancer stages (54). However, RHOB’s specific impact on ESCC is not fully clear. So, we explored RHOB’s biological functions in ESCC. Our results from the TCGA-ESCA and GSE53625 cohorts showed lower RHOB expression in ESCC tissues than in normal tissues, consistent with studies in ovarian, lung, stomach, and renal cell carcinoma (55–59). We used si-RHOB transfection to knock down RHOB in TE-1 and KYSE150 cells and confirmed it via qRT-PCR. CCK-8 assays revealed that RHOB knockdown significantly suppressed cell proliferation. Transwell assays demonstrated reduced migration and invasion of ESCC cells after RHOB knockdown, confirmed by wound - healing assays. Thus, RHOB promotes ESCC cell proliferation, migration, and invasion *in vitro*. In some studies, RHOB shows oncogenic properties by boosting cell proliferation and growth, reducing treatment responses, and leading to poor clinical outcomes, including primary tumor metastasis and short patient survival in colorectal cancer (60, 61). Conversely, RHOB exhibits tumor - suppressive properties by inhibiting cell invasion and metastasis and enhancing treatment responses, resulting in better clinical outcomes in bladder, ovarian, and head and neck cancers (62–64). These conflicting results may stem from differences in tumor types, animal and cell models, and research methods.

Our findings provide a novel framework for ESCC prognosis and clinical management, yet several limitations must be acknowledged. First, the sample size for scRNA-seq analysis was relatively small (n = 8), which may not fully capture the complete spatial and cellular heterogeneity of the ESCC immune landscape. Future studies with larger single-cell cohorts are required to refine these cell-state transitions. Second, our molecular subtyping was based on an *a priori* selection of genes related to CD8+ T cell differentiation rather than a genome-wide unbiased approach. While this ensures biological focus on T-cell evolution, it might overlook other critical genomic drivers of ESCC. Third, the therapeutic suggestions and drug sensitivity results in this study rely exclusively on in silico bioinformatic predictions. Although we utilized multiple algorithms to ensure robustness, these candidate compounds require validation in preclinical models and well-designed clinical trials. Finally, our investigation of RHOB was limited to *in vitro* functional assays without delving into its complex molecular mechanisms or using *in vivo* animal models. Further prospective, multicenter studies are essential to confirm the clinical utility of our identified molecular subtypes and the therapeutic potential of targeting the RHOB pathway.

## 5 Conclusion

In summary, three molecular subtypes of ESCC were constructed based on single-cell and bulk RNA sequencing. The relationships between the three molecular subtypes and prognosis, biological functions, genomic variation, and the immune microenvironment were revealed, and potential therapeutic drugs for the three molecular subtypes were explored. This provides new ideas to guide the personalized treatment strategy for patients with ESCC. In addition, Mendelian randomization analysis further indicated that genetically predicted elevation of RHOB expression significantly increase the risk of ESCC. *In vitro* cellular experiments also confirmed that knockdown of RHOB significantly inhibited the proliferation, migration and invasive ability of ESCC cells, providing experimental evidence to support the causal relationship identified by MR.

## Data Availability

The datasets presented in this study can be found in online repositories. The names of the repository/repositories and accession number(s) can be found in the article/**Supplementary Material**.

## Supplementary material

Supplementary Figure 1 Density plot of genes characterizing five immune cells. **(A)** Density plot of B cell signature genes, **(B)** density plot of plasma cell signature genes, **(C)** density plot of monocyte signature genes, **(D)** density plot of mast cell signature genes and **(E)** density plot of T cell signature genes.

Supplementary Figure 2 molecular isoforms constructed in the GSE53625 cohort. **(A)** NMF rank and phenotype correlation coefficients. **(B)** Heat map of the NMF consensus matrix. **(C)** Kaplan-Meier survival curves. **(D)** Multivariate Cox regression.

Supplementary Figure 3 Immune checkpoint molecular expression, immunotherapy and drug sensitivity analysis. **(A)** Comparative graph of the grouped expression of immune checkpoint molecules for the three molecular subtypes. Three molecular subtypes of **(B)** T-cell inflammatory signature score, **(C)** TIDE score, and **(D)** Exclusion score. IC50 box plots for **(E)** Olaparib, **(F)** Osimertinib, and **(G)** Rapamycin. ^ns^*P* > 0.05, **P* < 0.05,***P* < 0.01,****P* < 0.001.

Supplementary Figure 4 MR analyzes forest maps
