# Supplementary figures and images for: The construction of CD8+ T cell-associated molecular subtypes in esophageal squamous cell carcinoma reveals tumor heterogeneity, tumor microenvironment, and immunotherapy
Source: Front Immunol. 2026 Mar 27;17:1775837. doi: 10.3389/fimmu.2026.1775837 (PMC13066225; doi:10.3389/fimmu.2026.1775837)

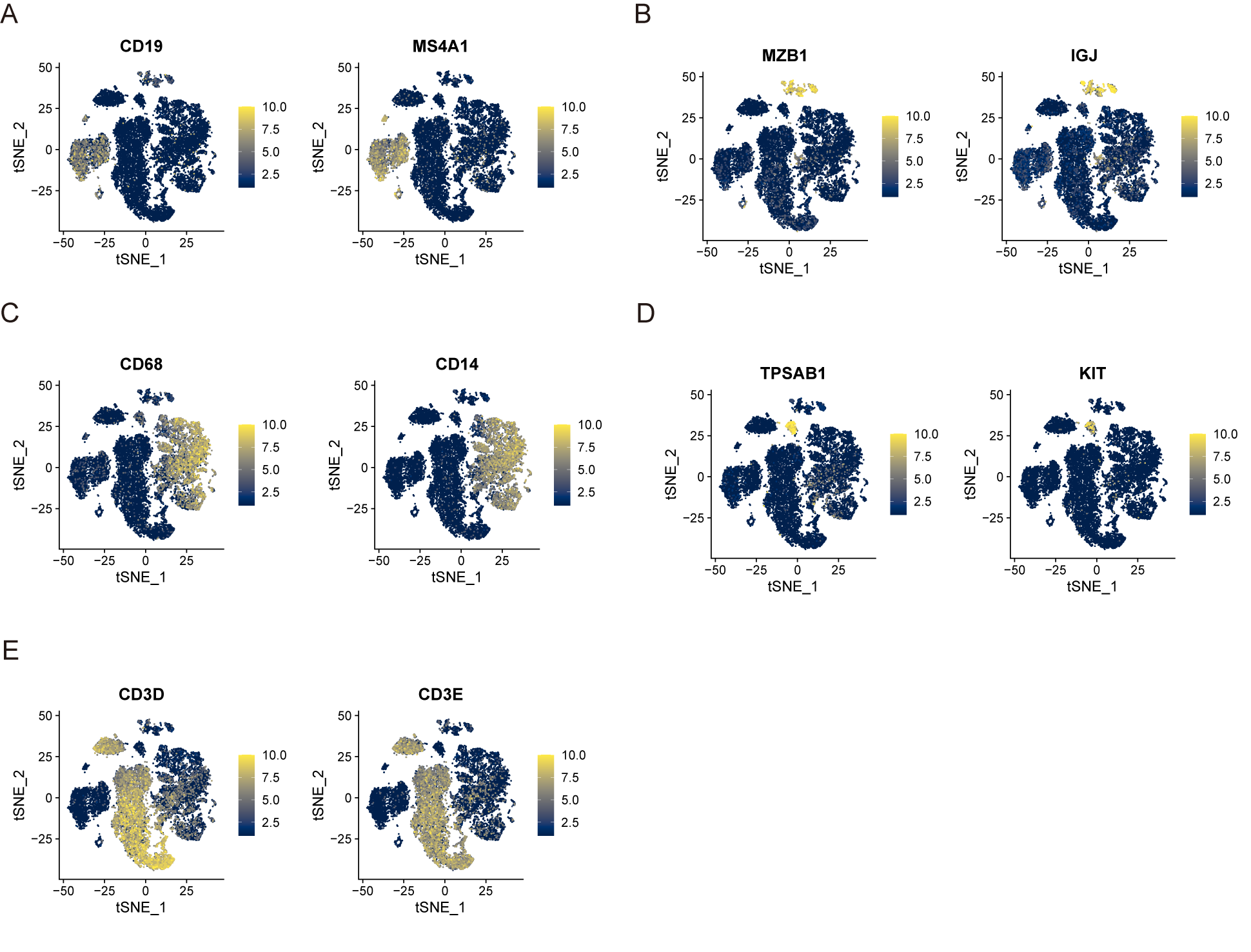

Supplement: Supplementary Figure 1 — Density plot of genes characterizing five immune cells. (A) Density plot of B cell signature genes, (B) density plot of plasma cell signature genes, (C) density plot of monocyte signature genes, (D) density plot of mast cell signature genes and (E) density plot of T cell signature genes. [file Image1.tif]

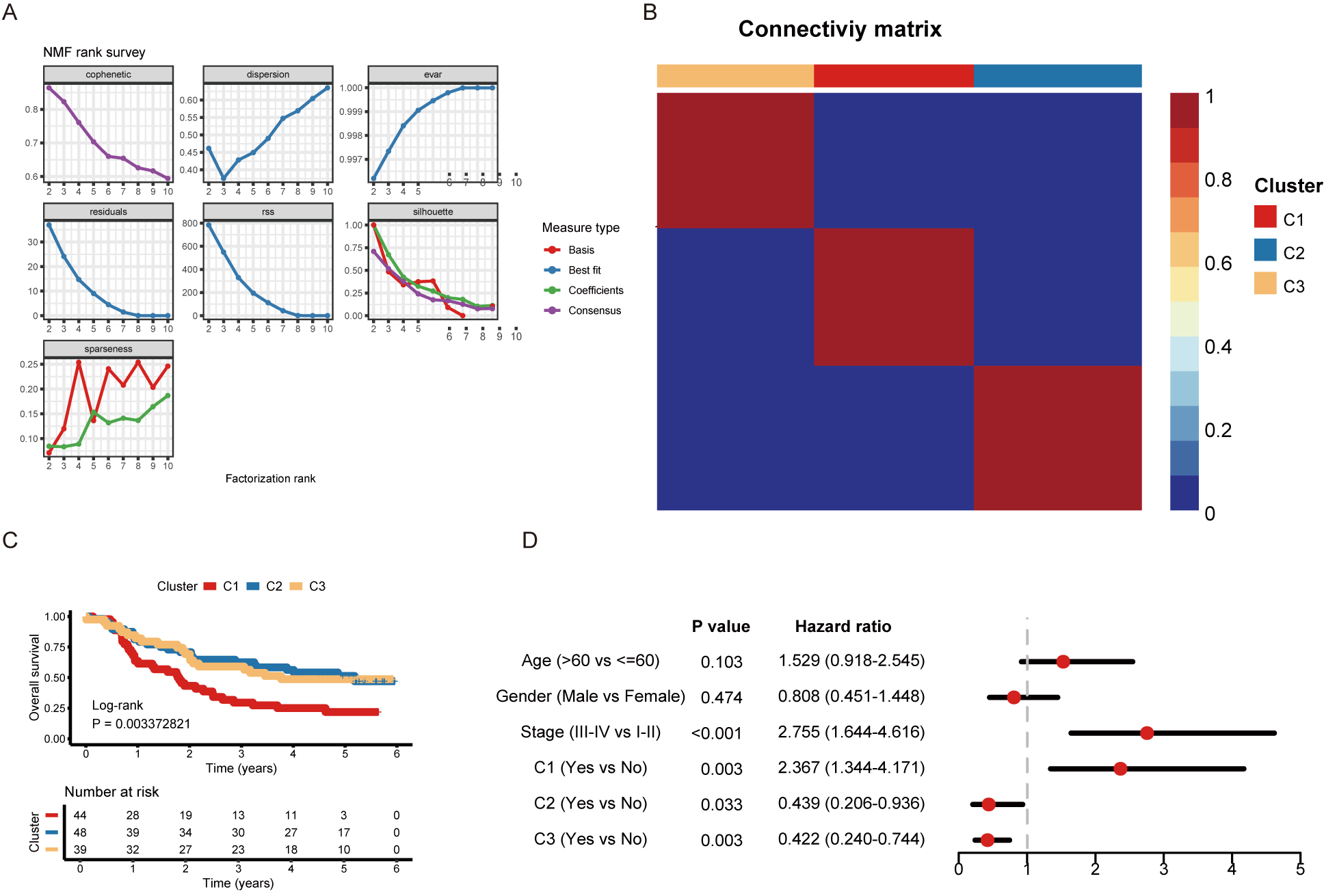

Supplement: Supplementary Figure 2 — molecular isoforms constructed in the GSE53625 cohort. (A) NMF rank and phenotype correlation coefficients. (B) Heat map of the NMF consensus matrix. (C) Kaplan-Meier survival curves. (D) Multivariate Cox regression. [file Image2.tif]

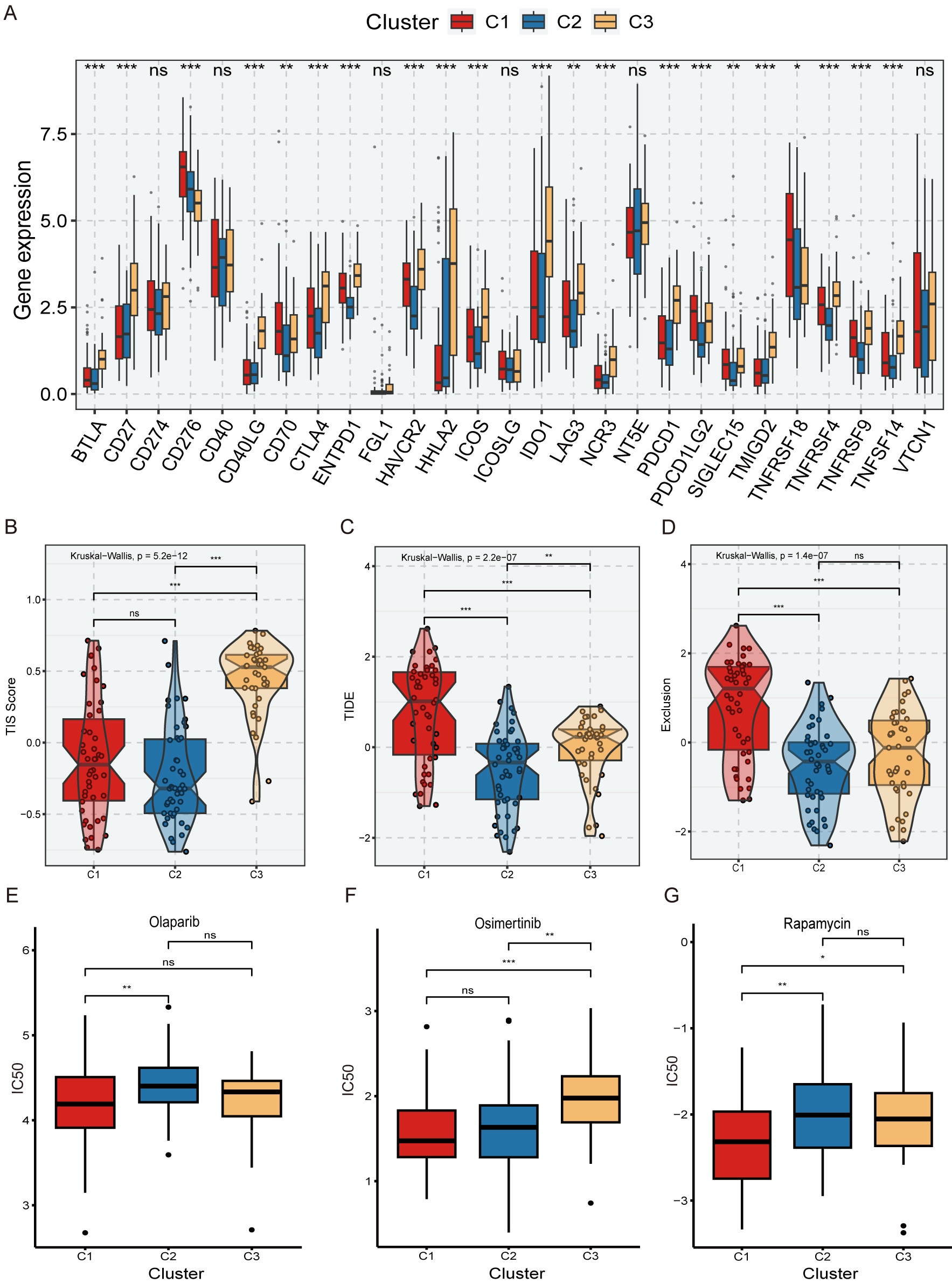

Supplement: Supplementary Figure 3 — Immune checkpoint molecular expression, immunotherapy and drug sensitivity analysis. (A) Comparative graph of the grouped expression of immune checkpoint molecules for the three molecular subtypes. Three molecular subtypes of (B) T-cell inflammatory signature score, (C) TIDE score, and (D) Exclusion score. IC50 box plots for (E) Olaparib, (F) Osimertinib, and (G) Rapamycin. nsP > 0.05, *P < 0.05,**P < 0.01,***P < 0.001. [file Image3.tif]

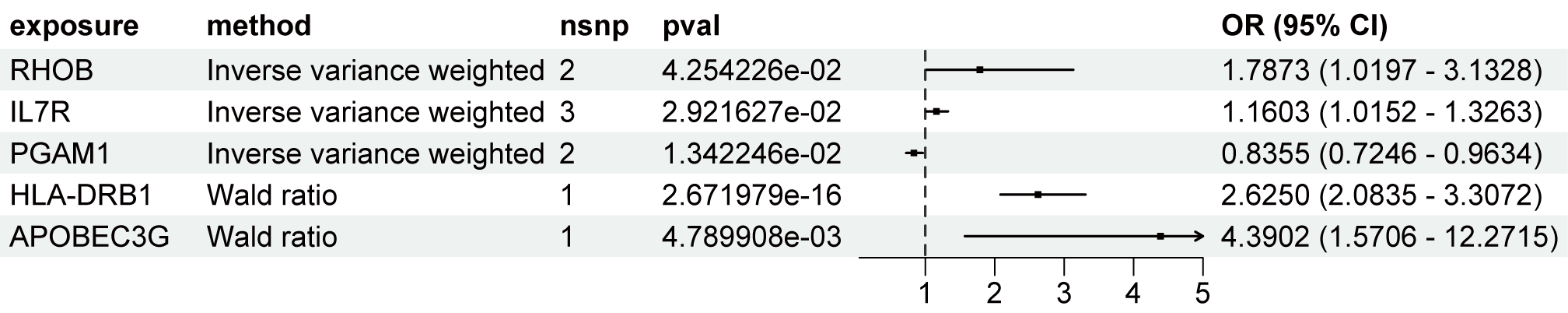

Supplement: Supplementary Figure 4 — MR analyzes forest maps [file Image4.tif]
